# Thoracic psammomatous meningioma with osseous metaplasia: a controversial diagnosis of a case report and literature review

**DOI:** 10.1186/s12957-019-1694-5

**Published:** 2019-08-24

**Authors:** Chao Wang, Yunqing Chen, Lu Zhang, Xuexiao Ma, Bohua Chen, Shuzhong Li

**Affiliations:** 1 Department of Spinal Surgery, the Affiliated Hospital of Qingdao University, 59, Haier Road, Qingdao, 266000 Shandong China; 2 Department of Pathology, the Affiliated Hospital of Qingdao University, Qingdao, Shandong China; 3 Department of Medical Research Center, the Affiliated Hospital of Qingdao University, Qingdao, Shandong China

**Keywords:** Meningioma, Psammomatous, Ossification, Thoracic spine, Metaplasia

## Abstract

**Background:**

Spinal psammomatous meningioma with calcification is commonly observed, but distinctive osseous differentiation rarely occurs.

**Case presentation:**

Here, we described a 52-year-old female complaining of chronic back pain for 5 years. CT and MRI examinations revealed an intradural extramedullary mass at the T4 level. The tumor was meticulously excised en bloc. Under the microscope, the tumor was found to be composed of conspicuous calcified psammoma bodies with remarkable immature bone formation. A primary diagnosis of psammomatous meningioma was made based on the recent WHO classification of tumors of the CNS, whereas other pathologists focused on the osseous components and preferred metaplastic meningioma as the proper subtype. A literature review was conducted, and only five cases have been reported with the same histopathological condition. Experts finally reached a consensus based on the acknowledged notion of the preferential diagnosis of psammomatous meningioma, as well as the current evidence and popular opinion that ossification is generated from osteogenic differentiation of pluripotent cells rather than the accumulation of psammoma bodies.

**Conclusions:**

A final diagnosis of psammomatous meningioma with osseous metaplasia was made. The rigid and adherent features complicate total resection of the tumor and increase the risk of neurologic deficits.

## Background

Although meningioma is a common benign neoplasm that accounts for 25–46% of spinal cord tumors, intralesional ossification rarely occurs [1]. When massive intralesional ossification occurs with the presence of conspicuous psammoma bodies, the exact pathological diagnosis is controversial. We searched papers definitely reporting both conspicuous ossification and psammoma bodies, and only five reports were included. For pathological diagnosis, Chotai et al. [9] and Prakash et al. [10] proposed psammomatous meningioma, while Licci et al. [8] and Chang et al. [3] preferred metaplastic meningioma. Therefore, it is necessary to discuss and clarify the best diagnosis for this special situation. Here, we presented a rare and similar case diagnosed as psammomatous meningioma with osseous metaplasia and elaborated on the reasons for this diagnosis.

## Case presentation

A 52-year-old woman presenting with a 5-year history of chronic back pain was seen at the outpatient center. Previously, the pain had been well controlled with massage and acupuncture, but the pain further deteriorated for 4 months before consulting a doctor and was occasionally induced even with a cough. Furthermore, the patient complained of moderate, intermittent, and painful numbness around the arch of the right rib and the left anterior thigh. She denied weakness and bladder or bowel dysfunction. On physical examination, interspinous tenderness was evoked at the T4–5 level without radical pain or paresthesia. Strength in the lower extremities as well as tendon reflexes was normal, and Babinski’s sign was negative bilaterally.

**Fig. 1:**
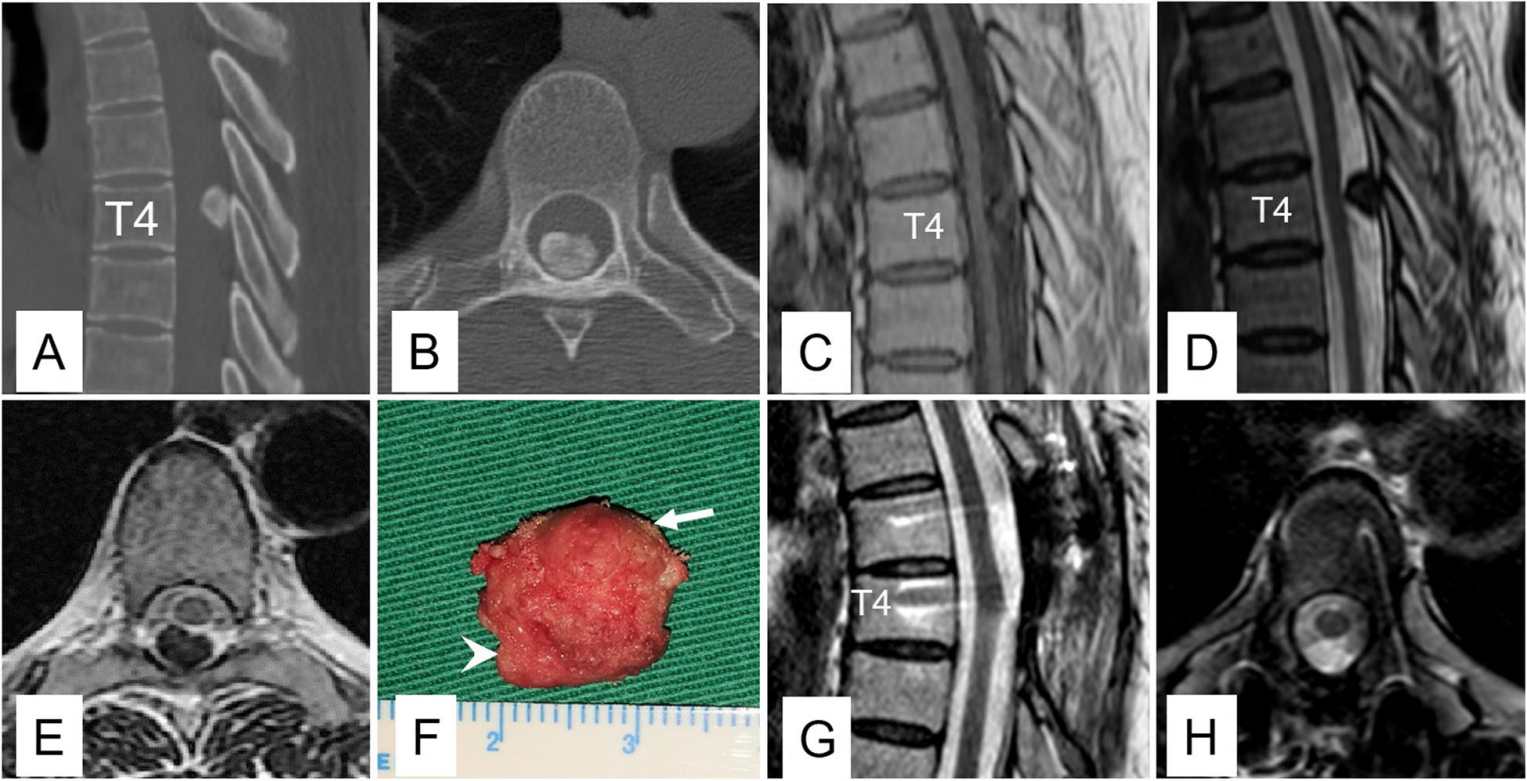
CT bone window (**a–b**) displayed an intraspinal tumor with high and uneven density on the dorsal side of the canal at the T4 level. Notice that there was an interspace between the neoplasm and lamina. MRI (**c–e**) revealed an intradural extramedullary mass, which pushed the spinal cord dorsally without obvious compression. The tumor showed hypointensity on both T1 and T2 images compared to the spinal cord. **f** Grossly, the tumor was a firm entity (arrow) with adhesion to the adjacent dura and arachnoid (arrowhead). Follow-up MRI images (**g–h**) showed no evidence of recurrence

Computed tomography showed an intraspinal tumor with high and uneven density on the dorsal side of the canal at the T4 level, with an interspace between the neoplasm and lamina (Fig. 1a, b). Magnetic resonance imaging (MRI) revealed an intradural extramedullary mass that pushed the spinal cord dorsally without obvious compression. The tumor showed hypointensity on both T1 and T2 images compared to the spinal cord (Fig. 1, c-e). According to the image characteristics, the preoperative diagnosis was meningioma.

Surgery was conducted after extensive discussion among the surgeons, and the goal was to remove the tumor safely and completely. A midline back incision was made from T3 to T5, and paravertebral muscles were detached subperiosteally and were softly distracted. After a laminectomy of T3 inferiorly, T4 in total, and T5 superiorly with an ultrasonic bone scalpel, the dural sac was exposed without adhesion to the lamina, which was consistent with preoperative imaging. Considering that the dura was rigid and calcified, a longitudinal spindle incision of the dura was made beyond the cephalic and caudal ends of the tumor, after which the tumor was peeled meticulously under spinal cord monitoring. Although there was little adhesion to the spinal cord, the tumor was tightly adhered to the adjacent arachnoid and dura. Finally, all the tissues were resected, and gross total resection was achieved (Fig. 1f). The patient recovered uneventfully, and she experienced significant improvement in back pain on the day she was discharged. At the 2.5-year follow-up, she experienced only residual numbness on the back, and a subsequent MRI scan showed no evidence of recurrence (Fig. 1g, h).

**Fig. 2:**
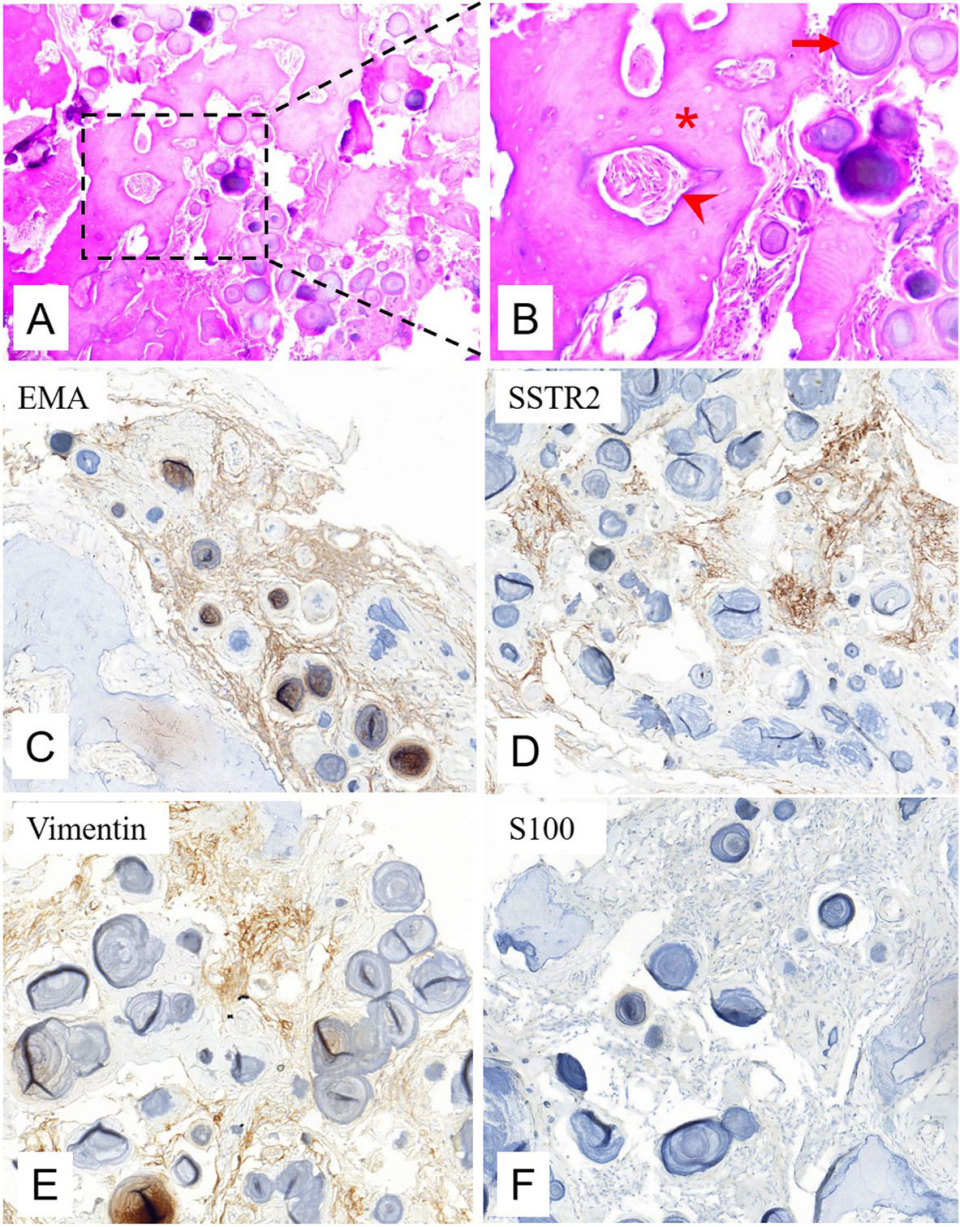
On H&E staining (**a** × 100; **b** ×  200), the lesion was found to be composed of numerous calcified psammoma bodies (arrow), with conspicuous immature trabecular bone formation (asterisk). Additionally, a rich blood supply (arrowhead) was observed in the foci of the bone component. The meningothelial cells were inconspicuous and lacked mitotic activity and necrosis. On immunohistochemical staining (**c–f**, × 100), the cells were positive for epithelial membrane antigen (EMA, **c**), somatostatin receptor 2 (SSTR2, **d**), and vimentin (**e**), but negative for S100 (**f**)

Grossly, the tumor was a firm entity measuring 1.2 × 1.1 × 0.9 cm, with adjacent dura and arachnoid (Fig. 1f). After 10% formalin fixation and decalcification and after being embedded in paraffin and stained with hematoxylin and eosin, the tumor was found to be composed of numerous calcified psammoma bodies under the microscope, with remarkable immature bone formation. Additionally, a rich blood supply was observed in the foci of the bone components, which indicated vigorous bone metabolism (Fig. 2a, b). The meningothelial cells were inconspicuous and lacked mitotic activity and necrosis. Upon immunohistochemical staining, the cells were negative for S100, but positive for epithelial membrane antigen (EMA), somatostatin receptor 2 (SSTR2), and vimentin, which are considered biomarkers of meningioma (Fig. 2c–f).

## Discussion and conclusions

The concept of ossification should be distinguished from calcification in meningioma, although these terms are misused occasionally in the literature [2–4]. Calcification is more of a radiological description than a histopathological diagnosis, and it is usually caused by an accumulation of numerous psammoma bodies and thus is commonly seen in psammomatous meningioma [3]. Ossified meningioma, which manifests as a mature or an immature bone texture within the lesion, is much rarer than calcified meningioma.

When it came to the pathological diagnosis, divergence emerged between the pathologists. The primary diagnosis was psammomatous meningioma based on remarkable psammoma bodies and the following description: “In psammomatous meningioma, the psammoma bodies often become confluent, forming irregular calcified masses and occasionally bone,” according to the recent WHO Classification of Tumors of the Central Nervous System [5]. In fact, one hypothesis is that psammoma bodies, which are much more common in spinal meningioma than in intracranial meningioma, can accumulate and assemble as new bone [6]. However, according to the same guidelines, other experts have emphasized the importance of osseous components and have suggested a “metaplastic meningioma” as the revised diagnosis, which is a subtype distinctive from psammomatous meningioma in the WHO classification; by definition, it is “a variant of meningioma with striking focal or widespread mesenchymal components including osseous, cartilaginous, lipomatous, myxoid, and xanthomatous tissue” [5].

To better understand this obscure issue, we searched the PubMed database with the following strategy: ((((((((ossified) OR calcified) OR osteogenic) OR osteoblastic) OR osseous) OR bone) AND spinal) AND meningioma). To eliminate any confusion with the presence of calcification, papers would not be adopted unless there was a definite pathological description of ossification. A total of 572 articles were retrieved. After screening the titles and abstracts and excluding irrelevant papers, 19 reports were identified. Finally, we focused on the five cases that involved conspicuous psammoma bodies and ossification (Table 1) [3, 7–10]. Similar to the diagnostic divergence found in our case, the diagnosis was not uniform. Two cases were diagnosed as psammomatous meningiomas, another two were diagnoses as metaplastic meningiomas, and the rest were diagnosed as meningiomas with simultaneous multiple subtypes. After a wide literature review, together with a multidisciplined discussion, we reached a consensus based on the following recognition and evidence.

**Table 1:** Diagnosis of spinal meningiomas with both conspicuous psammoma bodies and ossification

Year and authors	No. of cases	Summary of diagnosis
2009, Uchida et al. [7]	1	Psammomatous, fibrous, and metaplastic meningioma
2010, Licci et al. [8]	1	Psammomatous, fibrous, and metaplastic meningioma
2013, Chotai et al. [9]	1	Psammomatous meningioma with osseous metaplasia
2014, Chang et al. [3]	1	Metaplastic meningioma
2017, Prakash et al. [10]	1	Psammomatous meningioma with osseous metaplasia
Current case	1	Psammomatous meningioma with osseous metaplasia

First, admittedly, both psammomatous meningioma and metaplastic meningioma belong to WHO grade I, which is prone to a low risk of recurrence after total resection, whether en bloc or piecemeal [5]. Nevertheless, considering the abundance of intralesional psammoma bodies, a preferential designation of “psammomatous meningioma” seems more rational than “metaplastic meningioma,” which by definition should contain remarkable mesenchymal components rather than components predominant of other subtypes.

Second, even though the origin of osteogenesis in ossified meningioma remains unclear, most authors prefer to support that it is secondary to metaplasia of meningioma cells into osteoblasts, a process in which pluripotential stem cells could be involved [7, 11, 12]. Uchida et al. [7] speculated that ossification might be induced by exposure to biochemical activity of the ossification cascade or by secretion of osteoblast transforming factors such as Sox9 and Runx2 by mesenchymal premature cells. Metaplastic cells, which represent differentiated premature arachnoid cells, could be immunopositive for bone morphogenetic cytokines and the ossification process in meningiomas. Alafaci et al. [12] found that in 2 of 9 ossified meningiomas, in which no psammoma bodies were identified, foci with immature bone trabeculae and mineralized chondroid matrix were observed, suggesting a pattern of enchondral ossification. Lim et al. [11] successfully isolated what they called meningioma stroma mesenchymal stem-like cells (MS-MSLCs) in meningioma specimens, which could be one component of the meningioma cellular microenvironment, as well as a putative explanation for ossification in meningioma.

Finally, the theory of accumulating psammoma bodies that form bone has vulnerabilities within clinical observations. Although it is true that most ossified cases in the literature were accompanied by psammoma bodies, the ratios of osseous tissues and psammoma bodies are not proportional. In some reports, substantial ossification occurred with sporadic psammoma bodies [2, 13–17]. Moreover, several reports demonstrated obvious ossification but without psammoma body formation [12, 18–21].

Thereafter, the final diagnosis was psammomatous meningioma with osseous metaplasia. Total resection should be the first-line therapy. However, considering the compression and adhesion to the spinal cord due to the hard entity, especially when the tumor is located ventral to the spinal cord, the process of dissection and total resection is sometimes not a viable option. For our patient, as no symptoms or images of spinal cord compression were shown preoperatively, severe adhesion to the spinal cord was not expected. However, the adherent dura and arachnoid were removed for convenience during total resection, and electrophysiological monitoring, including SEP and MEP, was applied for the precaution of spinal cord injury. Other advanced techniques, such as the use of an ultrasonic bone scalpel or aspirator, are recommended for dealing with these thorny masses. Regular follow-ups for postoperative patients are extremely important, especially for those who receive subtotal resections.

In conclusion, we presented a case of subdural meningioma with conspicuous psammoma bodies and osseous metaplasia, and there have only been five similar reports in the literature. The final diagnosis was psammomatous meningioma with osseous metaplasia rather than metaplastic meningioma, which was based on several reasons. The rigid and adherent features complicate the total resection of the tumor and increase the risk of neurologic deficits.

## Data Availability

Not applicable.
